# Investigation of the efficacy of the short regimen for rifampicin-resistant TB from the STREAM trial

**DOI:** 10.1186/s12916-020-01770-z

**Published:** 2020-11-04

**Authors:** P. P. J. Phillips, A. Van Deun, S. Ahmed, R. L. Goodall, S. K. Meredith, F. Conradie, C-Y Chiang, I. D. Rusen, A. J. Nunn

**Affiliations:** 1 UCSF Center for Tuberculosis, University of California San Francisco, San Francisco, USA; 2 Leuven, Belgium; 3 MRC Clinical Trials Unit at UCL, London, UK; 4 Faculty of Health Sciences, University of Witwatersrand, Johannesburg, South Africa; 5 Division of Pulmonary Medicine, Department of Internal Medicine, Wanfang Hospital, Taipei Medical University, Taipei, Taiwan; 6 Division of Pulmonary Medicine, Department of Internal Medicine, School of Medicine, College of Medicine, Taipei Medical University, Taipei, Taiwan; 7 International Union against Tuberculosis and Lung Disease (the Union), Paris, France; 8 Research Division, Vital Strategies, New York, USA

**Keywords:** MDR-TB, Tuberculosis, Short regimen, Non-inferiority, Causal inference, Inverse probability of censoring weighting, Multiple imputation

## Abstract

**Background:**

The STREAM trial demonstrated that a 9–11-month “short” regimen had non-inferior efficacy and comparable safety to a 20+ month “long” regimen for the treatment of rifampicin-resistant tuberculosis. Imbalance in the components of the composite primary outcome merited further investigation.

**Methods:**

Firstly, the STREAM primary outcomes were mapped to alternatives in current use, including WHO programmatic outcome definitions and other recently proposed modifications for programmatic or research purposes. Secondly, the outcomes were re-classified according to the likelihood that it was a *Failure or Relapse (FoR) event* on a 5-point Likert scale: Definite, Probable, Possible, Unlikely, and Highly Unlikely. Sensitivity analyses were employed to explore the impact of informative censoring. The protocol-defined modified intention-to-treat (MITT) analysis population was used for all analyses.

**Results:**

Cure on the short regimen ranged from 75.1 to 84.2% across five alternative outcomes. However, between-regimens results did not exceed 1.3% in favor of the long regimen (95% CI upper bound 10.1%), similar to the primary efficacy results from the trial. Considering only Definite or Probable FoR events, there was weak evidence of a higher risk of FoR in the short regimen, HR 2.19 (95%CI 0.90, 5.35), *p* = 0.076; considering only Definite FoR events, the evidence was stronger, HR 3.53 (95%CI 1.05, 11.87), *p* = 0.030.

Cumulative number of grade 3–4 AEs was the strongest predictor of censoring. Considering a larger effect of informative censoring attenuated treatment differences, although 95% CI were very wide.

**Conclusion:**

Five alternative outcome definitions gave similar overall results. The risk of failure or relapse (FoR) may be higher in the short regimen than in the long regimen, highlighting the importance of how loss to follow-up and other censoring is accounted for in analyses. The outcome of time to FoR should be considered as a primary outcome for future drug-sensitive and drug-resistant TB treatment trials, provided sensitivity analyses exploring the impact of departures from independent censoring are also included.

## Background

Tuberculosis kills more people than any other infectious disease worldwide [1]. Disease with resistance to rifampicin is particularly difficult to cure; treatment regimens are longer with more toxic drugs than for drug-sensitive TB [2]. The STREAM trial evaluated a 9–11-month “short” regimen for the treatment of rifampicin-resistant TB and demonstrated non-inferior efficacy and comparable safety to the then current standard 20+ month “long” regimen [3]. The primary efficacy endpoint was a composite unfavorable outcome which included death, relapse, and treatment failure, in addition to treatment modifications for adverse events and poor adherence. The overall proportion of unfavorable outcomes was very similar between the two treatment arms in both modified intention-to-treat (MITT) and per protocol (PP) populations, 20.2% and 19.3% respectively for the long regimen and 21.2% and 18.1% respectively for the short regimen; HIV-adjusted differences (long minus short) of 1.0% (95% CI − 7.5%, 9.5%) and − 0.7% (95% CI − 10.5%, 9.1%) respectively. There were, however, some differences in the components of the composite outcome that merited further investigation; notably bacteriological unfavorable outcomes were more common on the short regimen whereas an unfavorable outcome due to loss to follow-up was more common on the long regimen. There are three additional motivations for the further analyses described herein.

Firstly, there is widespread recognition of the importance of secondary outcomes and supportive analyses in non-inferiority trials since standard approaches to analysis in superiority trials (particularly the intent-to-treat principle) can bias towards falsely declaring non-inferiority [4]. Both MITT and PP were pre-specified as co-primary analysis populations from the first version of the trial protocol in 2011 in line with guidance at the time [5–8]. A more recent commentary supports this approach [9], but other authors, however, recommend relegating a PP analysis to a secondary analysis [10]. There is no mention, for example, of a PP analysis in the 2016 FDA guidance on non-inferiority, although an “as-treated” analysis had been included in the earlier 2010 draft [4].

Secondly, different stakeholders or “consumers” of the results of randomized trials have different interests and therefore may find different ways of looking at treatment outcomes helpful. World Health Organization (WHO) treatment outcomes are intended for monitoring and reporting results from treatment programs [11]. An important alternative efficacy outcome, these have been the focus of WHO treatment guideline expert groups with the addition of post-treatment relapse [2, 12]. Phase III treatment trials for TB, however, continue to use a composite outcome similar to that in STREAM as the primary outcome for interpretation of results; examples include S31/A5349 (NCT02410772), STAND (NCT02342886), SimpliciTB (NCT03338621), and endTB (NCT02754765). Repairing this disconnect between clinical trials and guideline development is an important step to enhance the contribution of clinical trials data to global guidelines and policies.

Thirdly, unlike some infectious diseases, such as HIV or HCV where a viral load can be used to quantify treatment response in clinical trials [13], there is no definitive biomarker for TB disease that indicates whether actively replicating TB bacilli that cause clinical disease are still present in an individual’s body, although biomarkers of treatment response are in development [14]. “Cure” in a phase III trial must therefore be defined pragmatically as absence of disease at completion of treatment and continued absence after an adequate period of post-treatment follow-up. Treatment failure and relapse are often based on positive cultures on at least two consecutive occasions, or absence of culture conversion by end of treatment [15, 16]. The remaining participants, where these strict criteria for cure or failure are not met (comprising approximately 15–20% in recent trials [3, 15]), may or may not be cured and decisions as to how to consider them in the analysis has the potential to greatly affect the overall trial conclusions.

The objective of this secondary analysis from the STREAM trial was to further investigate the efficacy of the short regimen using two different broad approaches, and to provide guidance on the role and limitations of each approach for future TB clinical trials. The approaches were: 1) mapping the STREAM data to previously suggested alternative outcome definitions including WHO programmatic outcome definitions and other recently proposed modification for programmatic or research use, and 2) proposing an alternative method of analysis that focuses on the effect of the intervention on TB-specific failure and relapse events, considering the impact of informative censoring.

## Methods

STREAM Stage 1 was a non-inferiority randomized controlled trial. Participants were randomized in a ratio of 2:1 to a “short” 9–11-month regimen or the locally used standard of care “long” regimen that followed 2011 WHO guidelines for the treatment of Multi Drug-Resistant TB (MDR-TB) [3, 17]. Many pre-defined secondary efficacy outcomes have been reported [3] and online supplement [3], including time to unfavorable outcome and the intermediate outcomes of time to smear and culture conversion.

In this analysis, two different approaches were used to further investigate efficacy. The first was to map the STREAM outcomes (Favorable, Unfavorable or Not Assessable) to five alternatives in current use to explore their impact on the trial results.

The protocol-defined MITT analysis population was used for all analyses. This included all randomized patients with a positive culture at baseline, except for patients randomized in error, patients with isolates taken before randomization or up to week 4 that were subsequently found to be susceptible to rifampicin or resistant to both fluoroquinolones and second-line injectables on phenotypic drug susceptibility testing (DST). Patients with an outcome classified as Not Assessable and therefore excluded in the STREAM primary analysis were included in all the secondary analyses reported here.

The five current alternatives employed were:

A. *WHO drug-resistant TB (DR-TB) treatment outcomes* [11] (Table A2.2 in reference). These standardized definitions are intended for programmatic use to promote comparability of TB data between national TB programs and for monitoring of program performance. These end of treatment outcomes comprise cured, treatment completed, treatment failed, died, and lost to follow-up with the first two categories considered together as treatment success.
B. *Modified WHO DR-TB treatment outcomes that include an additional category of relapse after treatment success*, defined as bacteriological relapse after end of treatment cure [2, 12].
C. *TBNET proposed alternative to WHO outcomes that incorporate 1-year of post-treatment follow-up* [18]. These definitions seek to overcome limitations in the WHO outcomes where treatment success is largely driven by treatment completion rather than bacteriological results and where post-treatment data is not considered. Cure was defined as a negative culture status 6 months after treatment initiation, no positive culture thereafter, and no relapses within 1 year after treatment completion Follow-up in STREAM was only up to 132 weeks post-randomization, so a full year of post-treatment follow-up was not available for some patients on the long regimen when duration exceeded 80 weeks.
D. *Schwoebel et al. proposal for short DR-TB regimens* [19]. These definitions are intended for shorter DR-TB regimens, adapting the WHO outcomes which are implicitly intended for regimens of at least 18 months duration. These end of treatment outcome categories are the same as WHO DR-TB treatment outcomes with modified definitions for treatment failed and cured, mainly based on bacteriological responses.
E. *A STREAM pre-specified secondary efficacy endpoint in which outcomes were classified according to the patients’ culture status at week 132* regardless of treatment changes or intermediate culture results, similar to a simplistic intention-to-treat analysis. Further details are in the online supplement, Additional file 1.

A comparison of these outcomes is provided in Additional file 1:Table S1. Each of these five classifications were tabulated by treatment arm and the unadjusted difference in treatment success between arms calculated with 95% confidence intervals.

Our second approach to examining the efficacy of the short regimen was to focus on TB disease events and to re-classify each STREAM primary outcome according to the likelihood that it was a *Failure or Relapse event* on a five-point Likert scale: Definite, Probable, Possible, Unlikely, and Highly Unlikely. The protocol-defined MITT analysis population was also used for this analysis.

Failure or Relapse (FoR) events were envisaged as those that effective TB treatment should prevent, namely events resulting from disease that has not been adequately controlled and therefore requires treatment modification or re-treatment (excluding proven exogenous reinfection).

An event was considered *Highly Unlikely* to be an FoR event only if there was evidence of durable cure; this equated to the primary outcome classification of favorable which required completion of follow-up with negative cultures. A *Definite* FoR event required clear bacteriological evidence of failure or relapse (excluding a proven reinfection with exogenous strain of *Mycobacterium tuberculosis)*, a *Probable* FoR event required some evidence for failure or relapse (clinical, bacteriological, or radiological) in the absence of clear bacteriology (Table 1). The FoR classification was undertaken retrospectively by the authors with several rounds of refinement, but without consideration of treatment duration or allocated regimen.

**Table 1:** Mapping from primary outcome to FoR event

Likelihood classification as FoR event	Primary outcome classification, with further details where relevant for mapping	Total participants in MITT population
**Highly unlikely**	Favorable	292
**Unlikely**	Treatment change because of baseline DST results	3
	Treatment change because of investigator decision^a^	2
	Died during treatment or follow-up, culture converted when last seen, death not related to TB	13
	Treatment changed following proven reinfection with exogenous strain of M. tuberculosis (using whole genome sequencing or other appropriate method)	8
	Treatment change after adverse event	7
	Lost to follow-up after 76 weeks, culture converted when last seen	6
	Died within first 2 weeks of treatment, never achieved culture conversion	1
	Lost to follow-up before 76 weeks (but after 40 weeks), culture converted when last seen	4
**Possible**	Lost to follow-up before 76 weeks, patient withdrew consent^b^	8
	Treatment changed after patient withdrew consent for study medication^c^	4
	Died at 8 weeks having not yet achieved culture conversion, death not related to TB	2
	Treatment changed after loss to follow-up or poor adherence, with no positive bacteriology to suggest treatment failure	2
**Probable**	Died during treatment, probably related to TB	3
	Both positive and negative cultures within week 132 analysis window when last seen^d^	2
	Death 27 weeks after randomization, culture positive when last seen	1
	Relapse after treatment, signs and symptoms with limited bacteriology	1
	Reversion on treatment, signs and symptoms with limited bacteriology	1
**Definite**	Treatment changed following bacteriological reversion on treatment	14
	Treatment changed following bacteriological relapse after treatment	5
	Died following bacteriological reversion on treatment	2
	Lost to follow-up before 76 weeks following bacteriological reversion on treatment	1
	Treatment changed after failure to achieve culture conversion	1

^a^Treatment change so that participant could receive same treatment as young child (n = 1) or following a positive pregnancy test result (*n* = 1)

^b^Reason for withdrawal of consent was due to adverse event (*n* = 4), or reason unknown (*n* = 4). All but one withdrew consent during the intensive phase of treatment; the participant that was an exception was initially lost to follow-up from the intensive phase and subsequently returned and then withdrew consent

^c^Reason for withdrawal of consent was due to adverse event (*n* = 2), or reason unknown (*n* = 2)

^d^Results of m TB strain genotyping showed same strain as baseline (*n* = 1) and no comparison possible (*n* = 1)

The time to an FoR event was analyzed using the log rank test and Cox proportional hazards regression, where patients not experiencing an event were censored at the time of the censoring event which met criteria for Unfavorable or Not Assessable in the primary analysis. In the FoR analyses, the main groups of interest were those classified as having a Definite or Probable FoR event (with censoring of Possible, Unlikely and Highly Unlikely events), although sensitivity analyses were conducted considering different dichotomies.

Aside from the problem that a dichotomy of a 5-point ordered variable ignores important data, these analyses of time to an FoR event require the assumption of independent or non-informative censoring. This means that the likelihood of an FoR event at the time of censoring is assumed to be the same as for those in whom no censoring occurred. To account for the fact that this assumption of independent censoring may be inappropriate, we conducted two sensitivity analyses of time to FoR using inverse probability of censoring weights (IPCW) [20] and multiple imputation (MI) [21] respectively; the details are provided in the online supplement, Additional file 1.

## Results

### Alternative efficacy outcomes

**Fig. 1:**
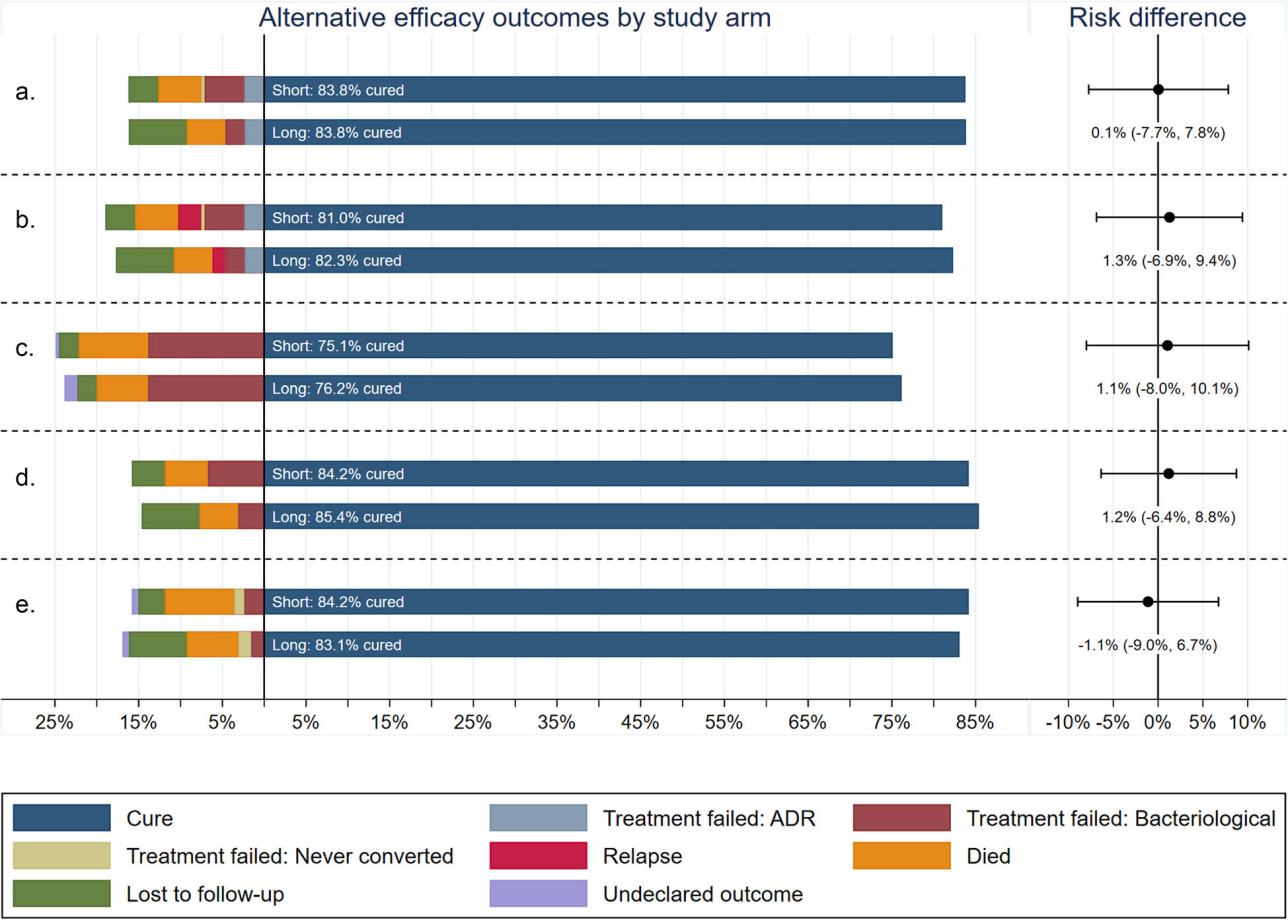
Summary of alternative secondary efficacy outcomes by treatment arm in MITT analysis population. Total length of bars is 100%; bars are centered at the cure dichotomy. (**a**) WHO end of treatment outcomes for rifampicin-resistant TB. (**b**) WHO outcomes modified to include post-treatment relapse. (**c**) TBNET MDR-TB outcomes [22]. (**d**) Modified WHO outcomes for short regimens [19]. (E) End of follow-up. Week 132 outcomes (week 132 outcomes: no culture after baseline is represented in the figure as “undeclared outcome”, culture positive at week 132 is represented as “treatment failed: bacteriological”, last culture positive prior to week 132 as “treatment failure: never converted” and last culture negative prior to week 132 as “lost to follow-up”)

Figure 1 and Additional file 1: Table S2 show the classification and results for each of the five alternative efficacy outcome definitions considered. Although the proportion with a classification of cure on the short regimen ranged from 75.1 to 84.2%, between-regimens results were similar to the primary efficacy results from the trial, not exceeding 1.3% in favor of the long regimen in any of the five classifications. The upper bound of the 95% confidence intervals did not exceed 10.1%.

### Time from randomization to failure or relapse event

**Fig. 2:**
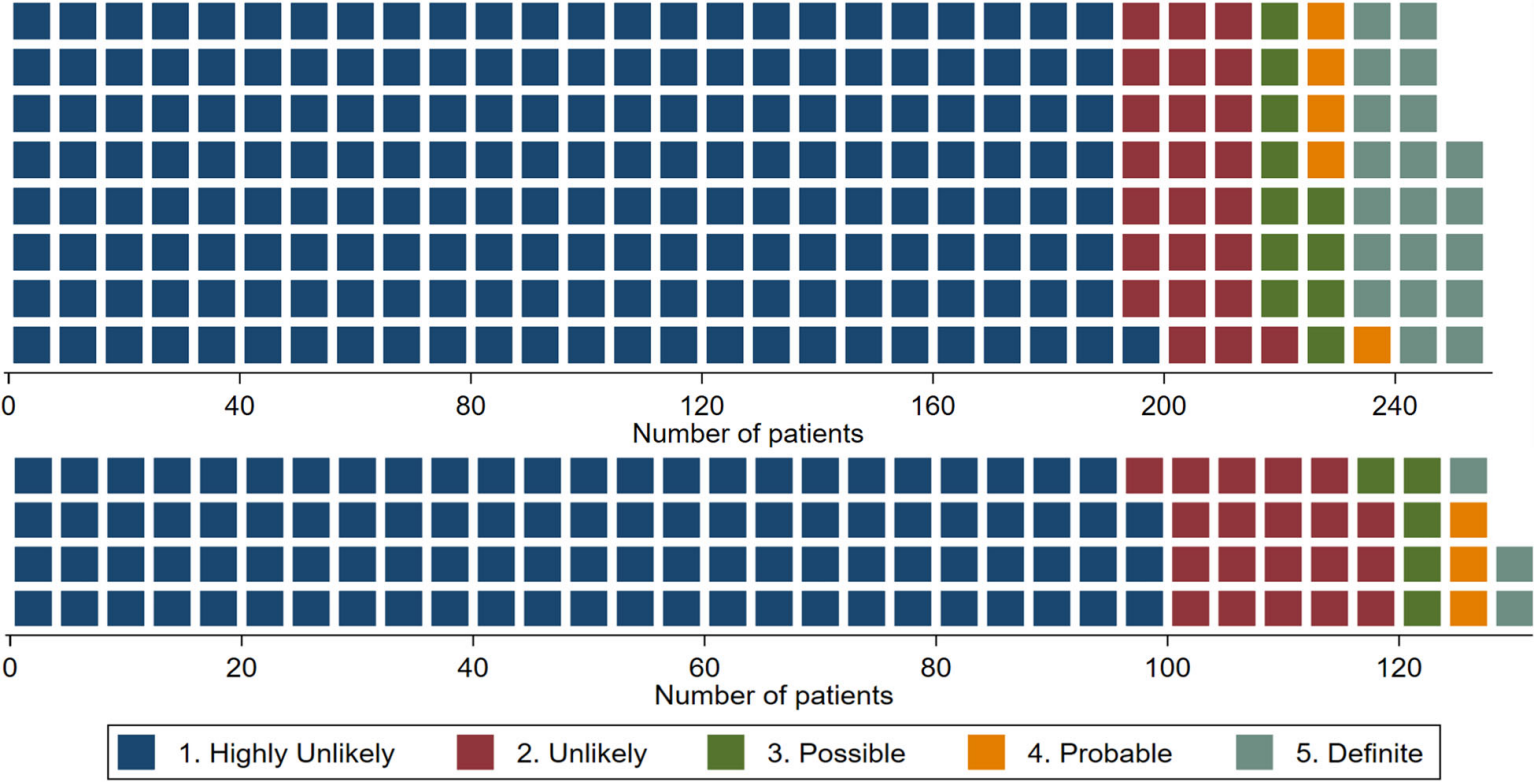
Summary of Failure or Relapse (FoR) event in MITT analysis population for short (upper) and long (lower) regimens. Each square represents a single patient; randomization was in a 2:1 allocation ratio in favor of the short regimen

**Fig. 3:**
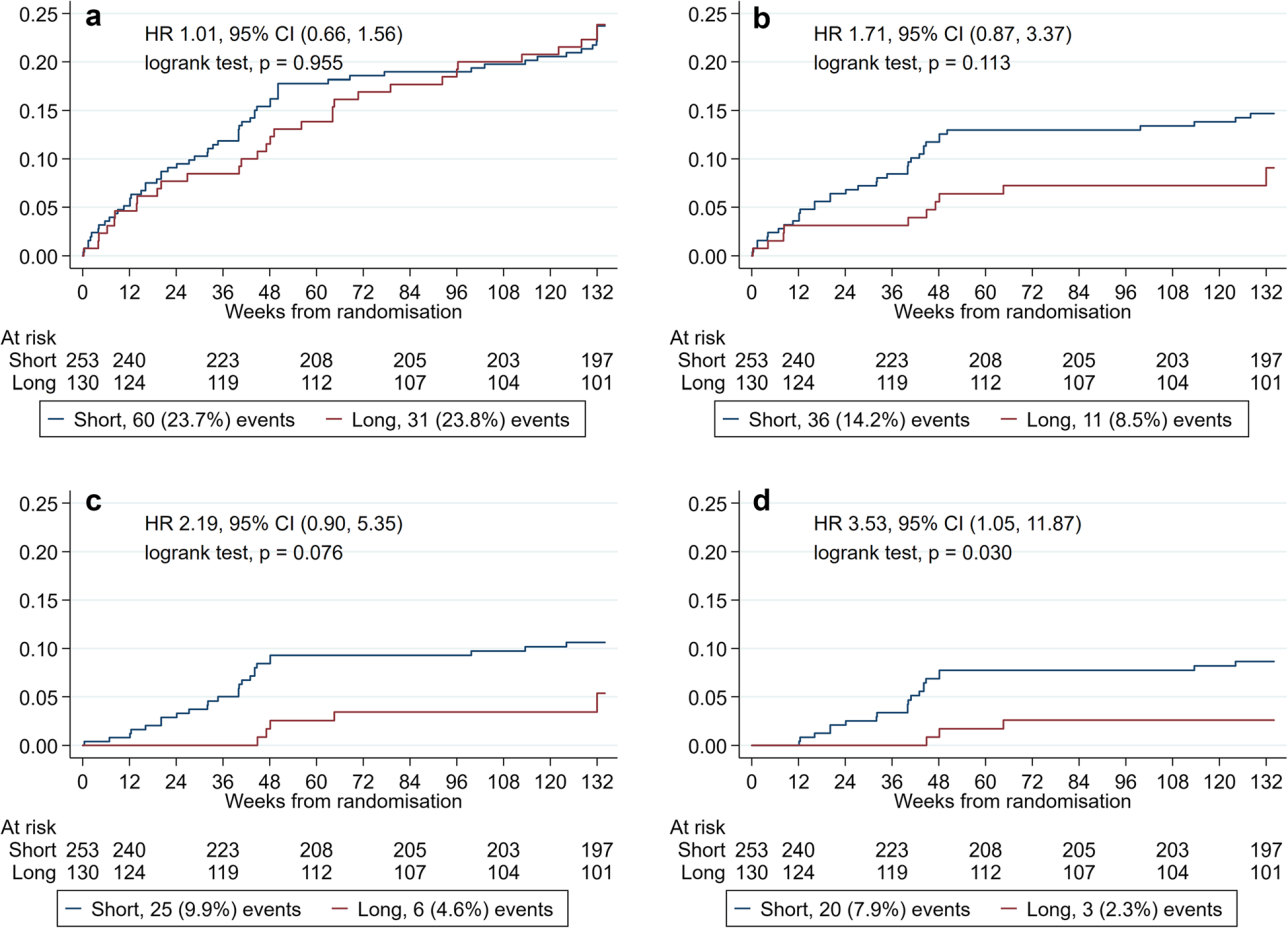
Time from randomization to Failure or Relapse event. An FoR event was defined as **a** Definite, Probable, Possible, and Unlikely as events; **b** only Definite, Probable, and Possible as events; **c** only Definite and Probable as events; and **d** only Definite as events

Figure 2 shows the breakdown of FoR events by treatment arm. When considering only Definite or Probable events, the confidence interval around the estimated hazard ratio was wide (Fig. 3c) with weak evidence of an increased risk of FoR among participants on the short regimen, HR 2.19 (95% CI 0.90, 5.35), *p* = 0.076. Including more categories decreased the hazard ratio estimate (Fig. 3b, a). When including only Definite events as FoR (Fig. 3d), there was evidence of a difference in time to FoR between arms, hazard ratio 3.53 (95% CI 1.05, 11.87), *p* = 0.030. No adjustment in the *p* values has been made for multiple comparisons.

**Fig. 4:**
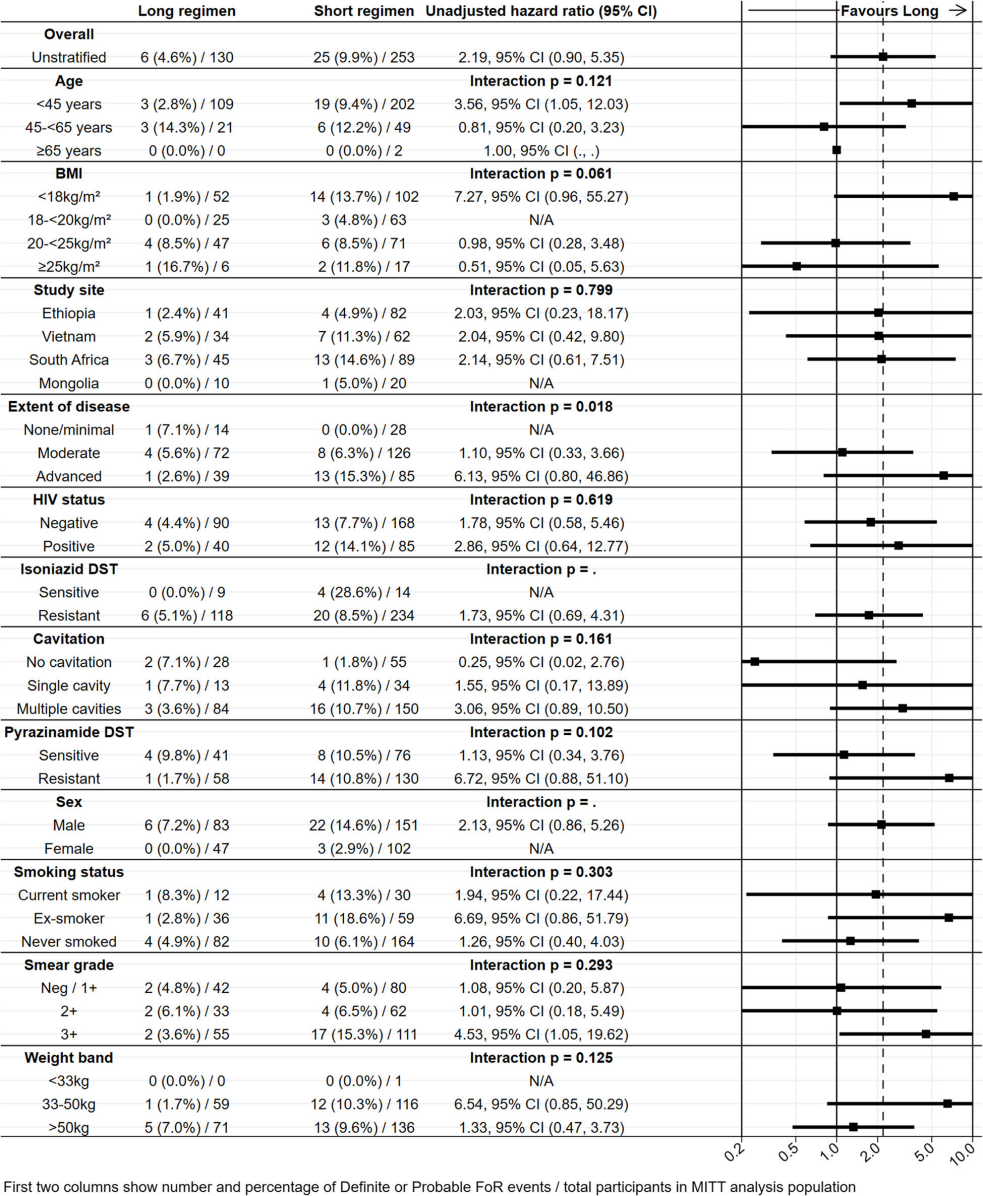
Forest plot of sub-group analyses for time from randomization to FoR event considering only Probable or Definite as events. There were no FoR events on the long regimen in female participants or participants with isoniazid-sensitive disease; no *p* value for the interaction test is therefore given for these comparisons

Considering the same subgroups which were evaluated in the primary STREAM publication, Fig. 4 shows a forest plot of subgroup analyses for FoR, defined as Probable or Definite events. Although the subgroup effects were slightly more pronounced than when using the primary outcome definitions [3], the only statistically significant interaction between subgroup and treatment is radiographic extent of disease, with more FoR events in participants on the short regimen with more advanced disease.

### Sensitivity analyses to account for informative censoring

The cumulative number of grade 3 and 4 AEs was the strongest predictor of censoring (Possible, Unlikely, Highly Unlikely FoR events) with higher odds of censoring with a greater number of AEs experienced prior to the event on both arms (Table 2). On the long regimen where more censoring occurred, censoring was also more likely if the most recent culture was positive, indicating that some of these censoring events may have masked true relapses thus supporting our sensitivity analyses exploring informative censoring. Table 3 shows the results of the IPCW analysis as compared to the unadjusted and adjusted analyses assuming independent censoring. The point estimate is slightly higher when including time-varying covariates and slightly lower without, but confidence intervals are wide across all analyses.

**Table 2:** Summary of predictors of probability of censoring (Possible, Unlikely, or Highly Unlikely FoR events) within time interval from logistic regression weight determining model. Table shows odds ratios and 95% confidence intervals from multi-variable logistic regression model. Odds ratios adjusted also for country of site and cubic spline (3 knots) of time-varying baseline hazard

Covariate	Level	Short regimen odds ratio (95% CI)	Long regimen odds ratio (95% CI)
Time varying: cumulative number of grade 3–5 AEs	0	Reference	Reference
	1	4.1 (1.2, 13.5)	6.2 (2.6, 15.2)
	2	13.3 (3.2, 54.9)	4.6 (1.4, 15.5)
	3	16.4 (2.8, 95.7)	11.5 (2.7, 50.0)
	4 or more	21.1 (3.3, 136.2)	28.7 (4.0, 204.9)
Time varying: most recent culture was positive		0.3 (0.0, 2.8)	3.4 (1.3, 8.6)
HIV positive at baseline		0.7 (0.2, 2.5)	1.9 (0.7, 5.4)
Baseline smear grading	Negative, Scanty, 1+	Reference	Reference
	2+	1.3 (0.4, 3.9)	0.5 (0.2, 1.3)
	3+	0.3 (0.1, 1.1)	0.2 (0.1, 0.6)
BMI at baseline, per 1 kg/m^2^		0.91 (0.77, 1.08)	0.87 (0.77, 0.99)
Age at baseline, per 1 year		1.01 (0.97, 1.06)	0.99 (0.96, 1.03)
Number of cavities on chest x-ray at baseline	None	Reference	Reference
	1	1.1 (0.2, 7.3)	0.4 (0, 3.1)
	2 or more	1.7 (0.5, 5.9)	1.2 (0.5, 3.2)

**Table 3:** Sensitivity analyses to account for informative censoring in time to FoR event. Definite and Probable included as events and Possible, Unlikely and Highly Unlikely considered censoring events

	Hazard ratio with 95% CI
**Unadjusted, assuming independent censoring**	2.19 (0.90, 5.35)
**Adjusted for baseline covariates, assuming independent censoring**	2.14 (0.87, 5.26)
**Adjusted, using IPCW with time varying covariates**	2.41 (0.92, 6.29)
**Adjusted, using IPCW with no time varying covariates**	1.96 (0.75, 5.14)

**Fig. 5:**
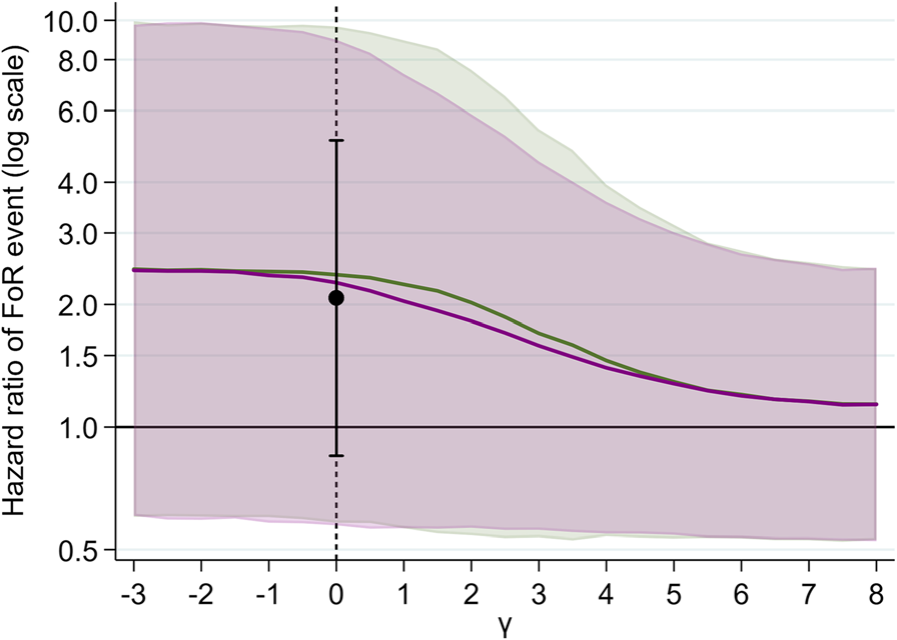
Estimate of hazard ratio (and 95% confidence region) for different values of *γ* after multiple imputation. *γ* was set to 0 (random censoring) for all Highly Unlikely events. In this analysis, *γ* represents the log hazard of FoR at the time of censoring as compared to another (covariate-matched) individual that is not censored. The green line (HR) and region (95% CI) corresponds to the assumption that the hazard ratio of an FoR event was double for Possible events as compared to Unlikely events; the purple line (HR) and region (95% CI) corresponds to the assumption that the hazard ratio of an FoR event was ten times for Possible events as compared to Unlikely events

Figure 5 shows the results of the multiple imputation analysis. Assuming a bigger effect of informative censoring (higher values of positive γ), corresponding to a higher hazard of FoR for a censored individual compared to an uncensored individual, gave smaller hazard ratios that were closer to 1.0 indicating a smaller between-treatment difference, although the confidence intervals were very wide. The slope of decline is slightly steeper when this hazard ratio comparing censored and uncensored individuals for Possible events is ten times that of Unlikely events (purple line) as compared to a doubling (green line).

## Discussion

In this paper, we have shown that cure proportions in both short and long regimens varied widely across alternative outcome definitions, but between-regimen differences did not. We have provided further evidence (albeit from post hoc analyses) suggesting that the hazard of failure or relapse (FoR) may be higher in the short regimen than in the long regimen. Further optimization of short DR-TB regimens to improve efficacy is urgently needed including dose optimization, the use of new drugs, and the use of alternative fluoroquinolones such as the novel delafloxacin [23] or gatifloxacin which, while unavailable now in many countries, has shown recent promise in MDR-TB observational cohorts [24]. These analyses highlight the importance of how loss to follow-up and other censoring are accounted for in analyses of clinical trial data.

These analyses of a new endpoint, time from randomization to a failure or relapse event (FoR), follow from a desire to better describe differences between regimens in terms of TB-specific efficacy while applying best practice for specification of estimands and analysis incorporating intercurrent events [25–27]. The standard survival Cox proportional hazards analysis, however, assumes that the chance of having a failure or relapse after a censoring event (had the event not occurred) is the same as for other participants in the study still in follow-up at that time point (the assumption of independent censoring [21]). This is, however, unlikely to be a reasonable assumption since those considered Possible or Unlikely include a variety of types of events that might be early indicators of failure or relapse such as poor adherence or withdrawal of consent (Table 1), and therefore, sensitivity analyses are paramount.

In our first sensitivity analysis, we employed the causal inference methodology of inverse probability of censoring weighting (IPCW [20]) to upweight uncensored individuals by the inverse of their probability of experiencing a censoring event. These individual-level probabilities are calculated based on baseline and on-treatment factors that are likely to predict the occurrence of a censoring outcome. The hazard ratio was slightly lower in this analysis, but with wider confidence intervals. More work is needed to explore implementation of this methodology to TB trial data, as is being done for TB observational data [28].

In our second sensitivity analysis, we imputed a time to failure or relapse event for individuals censored where this imputed time was predicted partly on baseline factors and partly on an explicit assumption as to how likely failure or relapse was to happen (the parameter γ) to see how sensitive our results were to this assumption [21]. We found that the difference in hazard of failure or relapse between arms was attenuated if we assume that a failure or relapse event was more likely to occur after a Possible or Unlikely FoR event than if these events had not occurred, represented by a positive γ. This means that analyses that fail to properly account for loss to follow-up and censoring by assuming independent censoring likely result in an under-estimate of the hazard of relapse and, in this trial, an over-estimate of the hazard ratio of failure or relapse between regimens. It should be noted, however, that confidence intervals are very wide, making precise determinations challenging, and we must assume a much higher chance of FoR event after censoring compared to no censoring in order for this effect to be non-negligible. For example, the hazard ratio of a FoR event between regimens is attenuated from about 2.0 to about 1.5 only when *γ* exceeds 4.0, corresponding to very high hazard ratio of FoR event between censored and uncensored observations of 55 for Unlikely and 110 for Possible events.

These analyses suffered from several limitations. Primarily, these post hoc analyses were not prespecified in the protocol or statistical analysis plan, and it was not possible to classify the likelihood of an event being a FoR event by a blinded independent committee since the primary trial results were already published. This might have introduced unconscious bias in classification or choice of methods for analysis; we therefore do not consider our FoR classification necessarily definitive for future trials. Ideally, the FoR classification and methods of analysis should be pre-specified and applied to trial data by a blinded independent endpoint review committee considering reasons for loss to follow-up and other censoring events. We would encourage future investigators to include all relevant stakeholders in the development of prospective consensus definitions for each type of event and intercurrent event in TB trials. As an example that could be replicated for TB trials, there are published descriptions of different types of AIDS-defining events [29] that have been used by blinded endpoint committees to adjudicate composite primary outcomes in large treatment trials in HIV such as START [30].

Grouping the FoR event into five categories is an improvement from a simple dichotomy as it permits sensitivity analyses but may be overly simplistic. Alternative approaches with more categories, or a continuous score, should be considered, as would analyses that preserve the categorical scale. We did not treat end of treatment failure separately from post-treatment relapse as we consider that this is not a straightforward dichotomy as shown by the number of bacteriological reversions occurring on treatment in the trial [3], although we acknowledge that the timing of failure and reversion may provide insight on the roles of different drugs in a regimen [31]. A further limitation was including only a limited number of baseline and time-varying covariates in the IPCW and MI models in the sensitivity analyses. Our relatively small sample size precluded extensive model development to identify the best predictors of censoring or FoR event. Our focus was on trials for new treatments for pulmonary tuberculosis; consideration of extra-pulmonary TB adds further complexity due to the challenge of collecting extra-pulmonary samples for smear or culture [1].

Alternative outcome definitions emphasize different aspects of treatment response that may be of interest to different stakeholders. For example, while there were more bacteriological failures observed on the short regimen, there were more patients lost to follow-up on the long regimen (as seen in other studies [32]); both are considered undesirable from a programmatic perspective which are reflected in outcomes intended for this. Nevertheless, programmatic outcome definitions are not well suited to the primary estimand and primary efficacy analysis of many randomized clinical trials where restrictions in eligibility and additional interventions to improve adherence to the protocol result in lost to follow-up and other treatment deviations that are unlikely to be representative of what might happen in a programmatic setting. Programmatic outcome definitions may, however, be suitable for the primary estimand for trials with explicit pragmatic designs, an example being the BEAT Tuberculosis trial evaluating a novel treatment strategy for all forms of rifampicin-resistant tuberculosis (ClinicalTrials.gov identifier NCT04062201).

Only data up to the end of treatment are used for the WHO outcome definitions (Outcome A.) which provides a very limited perspective in the STREAM trial as the median duration of treatment for patients that completed was 40.1 weeks (5th and 95th centiles 37.0, 46.3) for the short and 82.7 weeks (72.1, 102.3) for the long regimen [3]. Although including relapse in the WHO outcomes (Outcome B.) does mean that post-treatment bacteriology is included, cure is still defined at the end of treatment and therefore encompasses other post-treatment events that preclude identification of relapse (e.g., death or loss to follow-up). The TBNET outcomes (outcome C) were an improvement as a participant could only be included in the Cure category if they remained cured for 1 year after the end of treatment. However, the TBNET outcomes overestimate the number of failures in a clinical trial since only one positive culture is required, and isolated positive cultures in clinical trials with regular follow-up visits are a known phenomenon and do not necessarily indicate relapse and a need for further treatment [33, 34]. A modification that would overcome this limitation would be to require more than one positive culture for treatment failure (personal communication, Christophe Lange). Another limitation of the TBNET outcomes is that the period of follow-up is measured from end of treatment, and therefore, the total period of observation is longer for longer regimens, potentially biasing in favor of shorter regimens. For this reason, follow-up is recommended to be measured from randomization for all regimens irrespective of duration in TB clinical trials (see p200 of transcript from US FDA workshop [35]), even if this potentially biases in favor of the longer regimen, although there are differences of opinion. Longer post-treatment follow-up for shorter regimens may lead to more exogenous reinfection (although this can be excluded with whole-genome sequencing [36]) or loss to follow-up if patients lose interest after treatment completion, but this should be less of a problem in randomized controlled trials where loss to follow-up is minimized. The proposed modified WHO outcomes for short regimens (outcome D) were designed to be better suited to short regimens than the WHO outcomes, but suffer from the same limitations for clinical trials as they do not include post-treatment follow-up, although they do disaggregate efficacy and safety by removing adverse drug reaction as a cause of treatment failure. The week 132 outcomes (outcome E) show that a high number of patients, 84.2% and 83.1% in the short and long regimens respectively, were cured and had completed treatment at the end of follow-up, even if they previously had treatment failure or relapse and required changes or restart of treatment. This may be a useful supplementary endpoint for evaluating the impact of an intervention at a population level when considering a cascade of regimens approach [37] as it shows that TB disease can be cured at the end of two and a half years in a larger proportion of cases (provided there is no acquired drug resistance), even if retreatment or additional regimens are required.

## Conclusion

In conclusion, we believe time to failure or relapse event is an improvement on a simple dichotomous composite outcome and on analyses that exclude patients based on post-randomization data. This outcome should be considered as a primary outcome for future drug-sensitive and drug-resistant TB treatment trials, provided sensitivity analyses exploring the impact of departures from independent censoring are also included. We have shown further evidence (albeit from post hoc analyses) suggesting that the hazard of failure or relapse may be higher in the short regimen than in the long regimen pointing to the importance of further optimization of short DR-TB regimens to improve efficacy, including the use of new drugs.

## Supplementary information

**Additional file 1.** Contains supplementary methods describing the Week 132 outcome (alternative outcome E), a comparison of the five alternative outcomes (**Table S1**), and sensitivity analyses for the FoR analysis in more detail. Also includes supplementary results in **Table S2.** Summary of secondary efficacy outcomes by treatment arm in MITT analysis population.

## Data Availability

Individual patient data will become available in a publicly accessible repository. Please contact the corresponding author for more information.
