# Supplementary material for: Investigation of the efficacy of the short regimen for rifampicin-resistant TB from the STREAM trial
Source: BMC Med. 2020 Nov 4;18:314. doi: 10.1186/s12916-020-01770-z (PMC7640464; doi:10.1186/s12916-020-01770-z)
Supplement: Supplementary file 1 — Additional file 1. Contains supplementary methods describing the Week 132 outcome (alternative outcome E), a comparison of the five alternative outcomes (Table S1), and sensitivity analyses for the FoR analysis in more detail. Also includes supplementary results in Table S2. Summary of secondary efficacy outcomes by treatment arm in MITT analysis population. [file 12916_2020_1770_MOESM1_ESM.docx]

Further investigation of the efficacy of the short MDR-TB regimen: alternative analyses from the STREAM trial

Phillips PPJ, Van Deun A, Ahmed S, Goodall RL, Meredith SK, Conradie F, Chiang C-Y, Rusen ID, Nunn AJ

**Online Supplement**

**Supplemental Methods**

**Week 132 outcome (alternative outcome E)**

A participant was defined as “cured at Week 132” if the last two cultures were negative with the last no earlier than the Week 132 analysis window; other categories included death prior to Week 132, last culture positive at Week 132, last culture negative prior to Week 132, culture positive prior to Week 132, or no cultures after baseline.

**Sensitivity analyses for the FoR analysis**

The object of each sensitivity analysis is to explore the effect of relaxing the assumption of independent censoring in the FoR analysis. For each analysis, Definite and Probable were included as events where Possible, Unlikely and Highly Unlikely were included as censoring events.

In the first sensitivity analysis, we applied inverse probability of censoring weights (IPCW)(19). The IPCW analysis accounts for the missing data that occurred after censoring by increasing the ‘weight’ or ‘influence’ of data from comparable participants that were not censored in the analysis. Weights are calculated from a model for the probability of experiencing a censoring event, which included baseline covariates considered important in the primary analysis (HIV status, age, BMI, smear grade, country, and number of cavities) in addition to the time-varying covariates of cumulative number of severe (grade 3-4) AEs experienced and most recent culture result (positive or negative). Time during the trial was split into four-weekly intervals (to match the visit schedule from the STREAM Stage 1 trial(3)) and logistic regression used to model the probability of censoring within that interval. Separate models were used for each treatment arm. Stabilised weights were calculated as the ratio of the estimated probability of censoring from models without time-varying covariates and models with time-varying covariates(19). A pooled logistic regression model was used to estimate the ratio of hazards of a FoR event between arms adjusted for covariates and incorporating the stabilised weights. A cubic spline function of time was included in all models to account for a time-varying baseline hazard; knots were set at 16, 40, 78 and 104 weeks for the Short regimen and 26, 52, 78 and 104 for the Long regimen.

In the second sensitivity analysis, we used multiple imputation (MI) to impute the time of FoR for censored individuals(20) using the same baseline covariates for the imputation model as for the IPCW but no time-varying covariates. In addition, the multiple imputation model includes a parameter, γ, that reflects how much more likely we believe a FoR event is after censoring as compared to similar participants where censoring had not occurred. Since we may not have reliable information to estimate the likelihood of FoR for patients that are censored, we explore the impact on the overall results for a range of values. Using the same notation(20), the parameter γ, is the log of the hazard ratio of an FoR event, comparing a censored with an uncensored individual, both with the same baseline covariates. γ=0, for example, corresponds to independent censoring, γ=ln(5)=1.61 corresponds to the assumption that the hazard of FoR for a censored individual is 5 times that of an uncensored individual (with the same baseline covariates). In our analyses, γ was fixed at 0 for events considered Highly Unlikely, γ varied in the range (-3, 8) for events considered Unlikely, and was doubled (2γ) for events considered or Possible.

**Supplemental Results**

**Additional File 1: Table S1. Summary of five alternative outcomes, as compared to the primary outcome from the STREAM trial.**

|  | Successful / Favourable Outcome | | Unsuccessful / Unfavourable Outcome | | | |
| --- | --- | --- | --- | --- | --- | --- |
|  | **Cured** | **Treatment completed** | **Treatment failed** | **Died** | **Lost to follow-up** | **Not evaluated / Undeclared outcome** |
| STREAM Primary Outcome (ref. 3, online supplement) | ≥2 negative cultures, the last at 132 weeks after randomisation, no intervening positive culture, not unfavorable | N/ A | 1. Starting ≥2 new drugs, or 2. Treatment extension beyond the permitted duration, or 3. A positive culture from one of the two last specimens | Any death  ≤132 weeks after randomisation | Last seen ≤76 weeks after randomisation | N / A |
| A. WHO end of treatment outcomes for RR-TB (ref. 11) | Treatment completed as per national policy, without failure, AND ≥3 consecutive cultures (30 days apart) after IP | Treatment completed as per national policy, without failure, AND not cured | Permanent stop or change of ≥2 drugs because of:   1. No culture conversion by IP end, or 2. culture reversion in CP   after conversion, or   1. Acquired resistance to FQ or SLI, or 2. ADR | Any death during treatment | Treatment interruption for ≥2 consecutive months | No treatment outcome assigned (including transferred out, outcome unknown) |
| B. WHO outcomes modified to include post-treatment relapse | As with A., except when followed by post-treatment relapse | As with A., except when followed by post-treatment relapse | As with A., with the addition of post-treatment relapse | See A. | See A. | See A. |
| C. TBNET MDR-TB outcomes (ref. 17) | Negative culture status ≥6 months after treatment initiation, no later positive culture, no relapses ≤1 year after treatment completion | N/A | Positive culture status ≥6 months after treatment initiation or relapse ≤1 year after treatment completion. | Any death during observation | Non-receipt of care 6 months after treatment initiation. | Outcome not assessed (including transferred out, no culture status at 6 months, or no post-treatment data) |
| D. Modified WHO outcomes for short regimens (ref. 18) | Treatment completed as per national policy, without failure, AND ≥3 consecutive cultures (30 days apart) | Treatment completed as per national policy, without failure AND not cured | Positive culture after ≥6 months of treatment, except for isolated positive cultures (positive culture preceded by ≥1 and followed by ≥2 negative cultures) | See A. | See A. | See A. |
| E. End of follow-up Week 132 outcome | Culture negative status at 132 weeks after randomisation | N / A | Culture positive at 132 weeks after randomisation or culture positive when last seen <132 weeks. | Any death ≤132 weeks after randomisation | Culture negative status when last seen <132 weeks after randomisation | No culture results after randomisation |

Abbreviations: IP – Intensive Phase, CP – Continuation Phase, FQ – Fluoroquinolone, SLI – Second-line injectable, ADR – Adverse Drug Reaction

**Additional File 1: Table S2. Summary of secondary efficacy outcomes by treatment arm in MITT analysis population.**

|  | Study | Control | Total |
| --- | --- | --- | --- |
| Total in MITT Analysis Population | 253 | 130 | 383 |
| 1. ***WHO end of treatment outcomes for rifampicin-resistant TB (see reference 11)*** | | | |
| Cured | 212 (83.8%) | 109 (83.8%) | 321 (83.8%) |
| Treatment failed: Never converted | 1 (0.4%) | 0 | 1 (0.3%) |
| Treatment failed: Bacteriological reversion | 12 (4.7%) | 3 (2.3%) | 15 (3.9%) |
| Treatment failed: Adverse Drug Reaction (ADR) | 6 (2.4%) | 3 (2.3%) | 9 (2.3%) |
| Died on treatment | 13 (5.1%) | 6 (4.6%) | 19 (5.0%) |
| Lost to follow-up | 9 (3.6%) | 9 (6.9%) | 18 (4.7%) |
| 1. ***WHO outcomes modified to include post-treatment relapse*** | | | |
| Cured | 205 (81.0%) | 107 (82.3%) | 312 (81.5%) |
| Treatment failed: Never converted | 1 (0.4%) | 0 | 1 (0.3%) |
| Treatment failed: Bacteriological reversion | 12 (4.7%) | 3 (2.3%) | 15 (3.9%) |
| Treatment failed: Adverse Drug Reaction (ADR) | 6 (2.4%) | 3 (2.3%) | 9 (2.3%) |
| Relapse after Cure | 7 (2.8%) | 2 (1.5%) | 9 (2.3%) |
| Died on treatment | 13 (5.1%) | 6 (4.6%) | 19 (5.0%) |
| Lost to follow-up | 9 (3.6%) | 9 (6.9%) | 18 (4.7%) |
| 1. ***TBNET MDR-TB outcomes (see reference 17)*** | | | |
| Cure | 190 (75.1%) | 99 (76.2%) | 289 (75.5%) |
| Treatment failure | 35 (13.8%) | 18 (13.8%) | 53 (13.8%) |
| Death during observation | 21 (8.3%) | 8 (6.2%) | 29 (7.6%) |
| Loss to follow-up | 6 (2.4%) | 3 (2.3%) | 9 (2.3%) |
| Undeclared outcome | 1 (0.4%) | 2 (1.5%) | 3 (0.8%) |
| 1. ***Modified WHO outcomes for short regimens (see reference 18)*** | | | |
| Cure | 213 (84.2%) | 111 (85.4%) | 324 (84.6%) |
| Treatment failed | 17 (6.7%) | 4 (3.1%) | 21 (5.5%) |
| Died on treatment | 13 (5.1%) | 6 (4.6%) | 19 (5.0%) |
| Loss to follow-up | 10 (4.0%) | 9 (6.9%) | 19 (5.0%) |
| 1. ***End of follow-up Week 132 outcomes*** | | | |
| Favorable at Week 132 | 213 (84.2%) | 108 (83.1%) | 321 (83.8%) |
| Culture positive at Week 132 | 6 (2.4%) | 2 (1.5%) | 8 (2.1%) |
| Last culture positive prior to Week 132 | 3 (1.2%) | 2 (1.5%) | 5 (1.3%) |
| Died prior to Week 132 | 21 (8.3%) | 8 (6.2%) | 29 (7.6%) |
| Last two cultures negatives prior to Week 132 | 8 (3.2%) | 9 (6.9%) | 17 (4.4%) |
| No cultures after baseline | 2 (0.8%) | 1 (0.8%) | 3 (0.8%) |
| ***Failure or Relapse event*** | | | |
| Highly Unlikely | 193 (76.3%) | 99 (76.2%) | 292 (76.2%) |
| Unlikely | 24 (9.5%) | 20 (15.4%) | 44 (11.5%) |
| Possible | 11 (4.4%) | 5 (3.9%) | 16 (4.2%) |
| Probable | 5 (2.0%) | 3 (2.3%) | 8 (2.1%) |
| Definite | 20 (7.9%) | 3 (2.3%) | 23 (6.0%) |
